# Diversity of management strategies in Mesoamerican turkeys: archaeological, isotopic and genetic evidence

**DOI:** 10.1098/rsos.171613

**Published:** 2018-01-17

**Authors:** Aurelie Manin, Eduardo Corona-M, Michelle Alexander, Abigail Craig, Erin Kennedy Thornton, Dongya Y. Yang, Michael Richards, Camilla F. Speller

**Affiliations:** 1 BioArCh, Department of Archaeology, University of York, York, UK; 2 Centro INAH Morelos, Instituto Nacional de Antropología e Historia, Mexico; 3 Department of Anthropology, Washington State University, Pullman, WA, USA; 4 Ancient DNA laboratory, Department of Archaeology, Simon Fraser University, Burnaby, BC, Canada; 5 Department of Archaeology, Simon Fraser University, Burnaby, BC, Canada

**Keywords:** ancient DNA analysis, animal domestication, archaeology, isotope analysis, turkey (*Meleagris gallopavo*)

## Abstract

The turkey (*Meleagris gallopavo*) represents one of the few domestic animals of the New World. While current research points to distinct domestication centres in the Southwest USA and Mesoamerica, several questions regarding the number of progenitor populations, and the timing and intensity of turkey husbandry remain unanswered. This study applied ancient mitochondrial DNA and stable isotope (*δ*^13^C, *δ*^15^N) analysis to 55 archaeological turkey remains from Mexico to investigate pre-contact turkey exploitation in Mesoamerica. Three different (sub)species of turkeys were identified in the archaeological record (*M. g. mexicana*, *M. g. gallopavo* and *M. ocellata*), indicating the exploitation of diverse local populations, as well as the trade of captively reared birds into the Maya area. No evidence of shared maternal haplotypes was observed between Mesoamerica and the Southwest USA, in contrast with archaeological evidence for trade of other domestic products. Isotopic analysis indicates a range of feeding behaviours in ancient Mesoamerican turkeys, including wild foraging, human provisioning and mixed feeding ecologies. This variability in turkey diet decreases through time, with archaeological, genetic and isotopic evidence all pointing to the intensification of domestic turkey management and husbandry, culminating in the Postclassic period.

## 1. Introduction

Few animals have been fully domesticated in the New World. Among them, the North American wild turkey (*Meleagris gallopavo*) (hereafter referred to as the common turkey), represented an important resource in both Mesoamerica and the Southwest (SW) USA. Evidence of turkey bones and feathers recovered from multiple archaeological sites and contexts indicate that these birds played a significant role as food, raw material sources for bone tools and feather artefacts, as well as for medicinal and ritual purposes (e.g. [1–5]). Despite a growing body of literature concerning turkey exploitation in both regions [6–8], debate persists as to geographical origin of domestic turkeys in North America, the number of times the domestication process may have been initiated and how this process unfolded in both regions [9].

In the SW USA, ancient turkey husbandry began around 200–500 CE [4,10] and has been studied using a variety of analytical approaches, in particular osteological analyses (e.g. [11–14]), stable isotopes [7,15–18], genetics [7,19,20], morphometrics [21], palaeopathologies [22], coprolites [23] and eggshell characterization [15,24]. Stable isotopes, pathological features and pollen found in coprolites indicate that turkeys were usually not ‘free-range’, but often enclosed in pens or corrals, and provisioned with human staples, mostly maize. Whereas different domestic breeds have been claimed on the basis of osteological features and bone size [12], a strong sexual dimorphism, with smaller females than males, would explain most of the observed morphological variation [21]. Ancient mitochondrial DNA (mtDNA) analyses indicate that one maternal lineage predominates within the archaeological population of domestic turkeys from the Southwest that is distinct from that of the local Merriam's wild turkey (*M. g. merriami*) [19]. Although the geographical origin of the Southwest domestic lineage is still unclear, the predominance of a single maternal lineage indicates long-term continuity in turkey husbandry for over a millennium in this region.

In contrast to the American Southwest, historically far less research has been dedicated to uncovering the origins and timing of Mesoamerican turkey domestication, with bioarchaeological and morphometric approaches being applied relatively recently [25–30]. Within Mesoamerica, several questions concerning the domestication process remain unanswered, including the overall chronology of human intervention as well as the number of wild progenitor populations contributing to domestic stocks [9]. A recent synthesis of the Preclassic turkey remains (i.e. dating before 180 CE) suggests that common turkeys were already being captively reared during this period—albeit at a small scale—in different locations throughout Mesoamerica, including areas outside of their natural range [9]. The earliest confirmed evidence of captively reared common turkeys has been found in the Yucatan, at the site of El Mirador, Guatemala, dating from 327 BCE to 54 CE. Most recently, ancient DNA (aDNA) and isotope analysis (C, N, ^87^Sr/^86^Sr) has been used to identify specimens of common turkey, raised locally and provisioned with high quantities of C_4_ plants, probably maize [29,30]. Other Preclassic evidence, such as a complete turkey skeleton discovered in a sepulture in Oaxtepec, Morelos, México [31] or a representation of a turkey on an ocarina found in a grave of Monte Alban, Oaxaca [32] demonstrates the symbolic importance of this bird even in these early periods.

**Figure 1.**
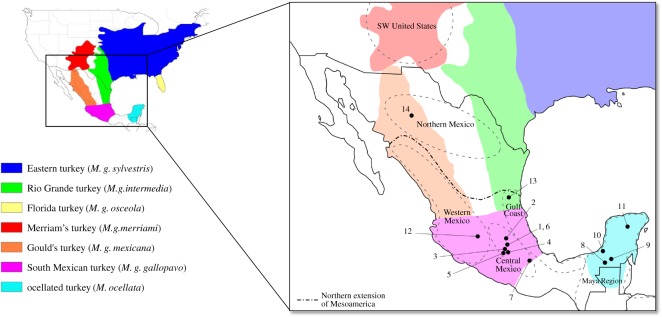
Historic distribution of North American wild turkey subspecies (*Meleagris gallopavo*) and the ocellated turkey *(Meleagris ocellata)* (after Schorger [5]) with the location of the sites analysed in this study and the regions mentioned in the text. 1, Texcoco (Estado de Mexico); 2, Teotihuacan (Estado de Mexico); 3, Terremote-Tlaltenco (Estado de Mexico); 4, Oaxtepec (Morelos); 5, Xochicalco (Morelos); 6, Huixtoco, Ixtapaluca (Estado de Mexico); 7, Santa Ana Teloxtoc (Puebla); 8, El Tigre (Campeche); 9, Calakmul (Campeche); 10, Champoton (Campeche); 11, Chichen Itza (Yucatan); 12, Malpais Prieto (Michoacan); 13, Vista Hermosa (Tamaulipas); 14, El Calderon (Chihuahua).

Several different regions have been proposed for the origin of domestication, including Western highlands of Michoacán [33], or the Mixteca of Oaxaca [34] but these assertions lack archaeological support. The wild progenitor subspecies also remains to be confirmed: while modern genetic analyses suggest that domestic turkeys were derived from the South Mexican wild turkey (*M. g. gallopavo*) [35], Mexico is home to two other wild populations, the Rio Grande wild turkey (*M. g. intermedia*) and Gould's wild turkey (*M. g. mexicana*), ranging within the Sierra Madre Occidental and Oriental, respectively (figure 1). It is unknown to what extent these populations may have been influenced by human intervention, and to what degree they may have contributed genetically to domestic stocks. In addition to the common turkey (*M. gallopavo*), the brightly plumed ocellated turkey (*M. ocellata*) is found within Mexico's Yucatan peninsula, and parts of northern Belize and Guatemala (figure 1). While ocellated turkeys are not currently raised as domestic birds in the Yucatan, their potential captive rearing has long been discussed among zooarchaeologists and ornithologists, although this practice has not been conclusively demonstrated in the archaeological record [30]. Finally, previous aDNA research suggested that the domestic turkey of the SW USA was genetically distinct from the Mesoamerican lineage [19] with no evidence for trade of domestic turkey between the two regions. No genetic data, however, have been obtained from ancient Mesoamerican turkey to explicitly test this hypothesis.

Our study aimed to elucidate the geographical origins and wild progenitors of Mesoamerican turkeys, and document the nature, intensity and chronology of human intervention in Mesoamerica through biomolecular analysis of archaeological turkey remains. Here, we applied ancient mtDNA analyses to 55 archaeological turkey remains to explore both phylogeographical patterning and the level of human intervention in turkey reproduction and breeding. We also applied stable isotope (*δ*^13^C, *δ*^15^N) analysis of bone collagen to explore human provisioning and restrictions on turkey diets. We contrasted these biomolecular data with archaeological evidence of distribution and abundance of turkey remains through time and space to investigate the geographical origins, timing and intensity of Mesoamerican turkey domestication.

## 2. Material and methods

### 2.1. Archaeological turkey bones

The archaeological turkey samples analysed in this study were obtained from the Instituto Nacional de Antropología e Historia, Mexico City (INAH), the Facultad de Ciencias Antropológicas, Universidad Autónoma de Yucatán (UADY), the Instituto de Investigaciones Antropológicas, Universidad Nacional Autónoma de México (UNAM), the Centro de Estudios Mexicanos y Centroamericanos (CEMCA) and the University of Calgary (UofC). A total of 55 turkey bones from 14 different archaeological sites in Northern Mexico, Central Mexican Highlands, Western Mexico, the Gulf Coast and Yucatan were analysed, including seven archaeological specimens previously analysed using aDNA by Speller *et al*. [19]. Electronic supplementary material, table S1 lists the provenance and element information for the samples, and the site locations are depicted in figure 1. Prior to biomolecular analysis, samples were photographed and measured according to criteria outlined in Von den Driesch [36]. Of these 55 bones, 53 were large enough to supply material for isotopic and genetic analyses.

### 2.2. Radiocarbon dating

Five samples were sent for radiocarbon dating at the Center for Applied Isotope Studies at the University of Georgia to validate the chronology. Sample preparation methods and main results are outlined in electronic supplementary material, Material 1, and the results are presented in table S2.

### 2.3. Stable isotope analysis

Fifty-three bones were prepared for isotopic analyses at the Department of Anthropology, University of British Columbia (Vancouver, Canada) and at BioArCh, Department of Archaeology, University of York, (York, UK). Collagen was extracted by following the procedure outlined in electronic supplementary material, Material 1. The results are expressed in per mil (‰) in reference (*δ*) with established standards (Vienna Pee Dee Belemnite (VPDB) for *δ*^13^C and atmospheric air (AIR) for *δ*^15^N). All samples produced acceptable C : N ratios (between 2.9 and 3.6, [37]) %C and %N values [38], and the errors on the *δ*^13^C and *δ*^15^N measurements are less than 0.2‰ (electronic supplementary material, table S1). As the ultrafiltration step can significantly reduce the amount of collagen retrieved [39], the threshold of 1% collagen yield has not been used, although yields are reported.

Statistical analyses of the isotopic data were conducted using PAleontological STatistics software (PAST, v. 3.08; [40]), with *p* values < 0.05 considered significant. Initial assessment of the data through the use of a Shapiro–Wilk test rejected the hypothesis of normal distribution for both *δ*^13^C (*W* = 0.8, *p* < 0.001) and *δ*^15^N (*W* = 0.9, *p* = 0.03), and only non-parametric tests were applied to identify differences between groups. Overall variability between more than two groups was assessed by Kruskal–Wallis tests. Mann–Whitney tests for equal median were used to compare groups, integrating a Bonferroni correction when more than two groups were involved. For each group, mean and standard deviation have been calculated and the values are expressed with a precision of ± 1 s.d. As isotopic data can be considered to be continuous values, correlation between *δ*^13^C and *δ*^15^N was calculated using the Pearson method.

### 2.4. Ancient DNA analysis

Of the 55 turkey bones included in the study, seven had been previously analysed in Speller *et al*. [19]; the remaining 48 samples of archaeological turkey bones were analysed in three ancient DNA laboratories (electronic supplementary material, table S1), located in the Department of Archaeology, Simon Fraser University (SFU), the Department of Anthropology and Archaeology, University of Calgary (UofC), and BioArCh, University of York (UofY). In all three laboratories, sample preparation and DNA extraction followed the silica spin-column protocol [41] modified as described in Speller *et al*. [19] (see electronic supplementary material, Material 1 for detailed methods). Overlapping sequences were concatenated and truncated to 438 bp (position 15 567–16 004, based on the complete mtDNA genome of GenBank specimen NC010195) to remove primer sequences, and make them comparable to reference sequences found in Mock *et al*. [42] and Speller *et al*. [19]. The obtained D-loop haplotypes (submitted to GenBank under Accessions: MF947187-MF947219) were compared with 298 *Meleagris* GenBank entries, including modern commercial breeds [35] and North American wild turkeys [42,43] as well as the 12 ancient haplotypes identified in the archaeological turkey remains recovered from the American Southwest [19] using ClustalW [44] through BioEdit [45] and Network (v. 5.0) [46].

## 3. Results

### 3.1. Species identification and phylogenetic analysis

We recovered mitochondrial DNA from 33 of the 48 bones, an overall success rate of 69% (table 1). Twenty samples yielded the entire 438 bp fragment, while an additional 13 samples yielded partial mtDNA profiles, sufficient for species identification, and in some cases for haplotype identification (electronic supplementary material, table S1). The majority of the turkey bones (*n* = 31) were identified as *M. gallopavo* and two as *M. ocellata*. Both *M. ocellata* samples were recovered from the site of Calakmul, Yucatan, within the natural range of the ocellated turkey. The 31 *M. gallopavo* samples were more widely distributed, with 29 individuals recovered from archaeological sites within the natural distribution of the common turkey, and four individuals identified at the Postclassic Yucatan site of Champotón. Four different *M. gallopavo* haplotypes were identified in the remains: 14 turkeys were identified as mHap1, 12 as mHap2, 1 as mHap2a and 2 as mHap2b.

**Table 1.** Summary of the genetic identification per region. The number of specimens indicated here only takes into account the specimens with positive genetic identification. Samples from Northern Mexico were analysed in [19].

geographic area	no. specimens	(sub)species and haplotypes identified	local (sub)species
Northern Mexico	2	*M. g. mexicana* (aHap2e)	*M. g. mexicana*
Western Mexico	5	*M. g. gallopavo/intermedia* (mHap1)	*M. g. gallopavo*
Gulf Coast	7	*M. g. gallopavo/intermedia* (mHap2, mHap2b)	*M. g. intermedia*
Central Mexican Highlands	15	*M. g. gallopavo/intermedia* (mHap1, mHap2, mHap2a, mHap2b)	*M. g. gallopavo*
Yucatan Peninsula	6	*M. g. gallopavo/intermedia* (mHap1, mHap2); *M. ocellata*	*M. ocellata*

The two most common haplotypes identified in the archaeological remains, mHap1 and mHap2 (as described in Speller *et al*. [19]) have been previously observed in modern domestic turkeys, Rio Grande wild turkeys (*M. g. intermedia*) and the historic specimens of South Mexican wild turkey (*M. g. gallopavo*); haplotypes mHap2a and mHap2b (differing from mHap2 by one base pair, respectively) have not been previously reported in modern domestic or wild turkey. Archaeological sites within Central Mexico displayed the greatest diversity with the presence of all four haplotypes. Haplotypes mHap1 and mHap2 were observed in archaeological specimens from Yucatan (*n* = 1, respectively), while Western Mexican turkeys were identified only as mHap1 (*n *= 5), and Gulf Coast turkeys were identified only as mHap2 (*n* = 3) or mHap2b (*n* = 1).

**Figure 2.**
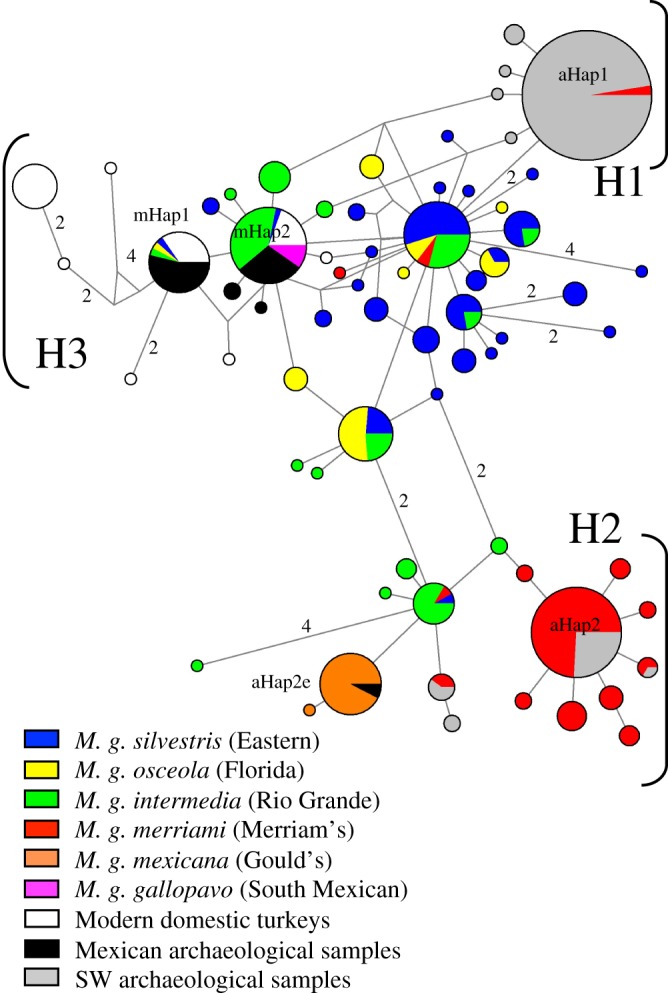
Median-joining network displaying the relationships between the obtained sequences and existing archaeological [19] and modern [35,42,43] sequences, obtained from GenBank. Mexican turkeys (in black) are compared with archaeological samples from the SW USA (in grey), modern breeds (in white) and wild subspecies (according to the colours). Mesoamerican samples are primarily grouped in the mHap1 and mHap2 haplotypes, whereas North Mexican individuals fall within the aHap2e haplotype.

When phylogenetically compared with other wild and domestic turkey haplotypes, the Mesoamerican archaeological turkey samples fall into clade H3 (as described in Speller *et al*. [19]) including modern European domestic turkey breeds [35], North American commercially raised birds, as well as the limited sequences available from historic specimens of the South Mexican wild turkey (*M. g. gallopavo*), the local wild turkey of South Central Mexico (figure 2). None of the samples we extracted in this study had haplotypes observed in Gould's wild turkey (*M. g. mexicana*), although this subspecies was previously detected in the archaeological samples analysed from the Calderón site in Northern Mexico [19], which we analysed using isotopic analysis. Likewise, we did not detect the dominant haplotype observed in the domestic turkeys of the American Southwest (aHap1) in any of the ancient Mesoamerican turkey bones.

### 3.2. Stable isotope analyses

Our data indicate a range of feeding behaviours that were undertaken by turkeys, including diets demonstrating a reliance on C_3_ plants (*δ*^13^C of approx. −22 to −19‰), diets dominated by C_4_ plants (*δ*^13^C approx. −10 to −6‰) and a mixed feeding ecology (*δ*^13^C approx. −18 to −11‰) [47,48] (table 2). Table 2 presents a summary of the results of isotope ratios; individual values, as well as collagen quality and yields are described in electronic supplementary material, table S1. Collagen yields ranged from 0.2 to 11.6% per sample, with elemental carbon ranging from 37.6 to 44.2% and elemental nitrogen ranging from 13.5 to 16.0%. Overall, *δ*^13^C values ranged between −20.5 and −6.6‰ (mean *δ*^13^C = −11.8 ± 4.60‰, 1 s.d.), and *δ*^15^N values ranged between 3.5 and 9.8‰ (mean *δ*^15^N = 7.3 ± 1.65‰, 1 s.d.). There is a positive correlation between carbon and nitrogen ratios (Pearson's *r* = 0.71, *p *< 0.001), indicating that individuals with higher *δ*^13^C values tend to have corresponding higher *δ*^15^N values.

**Table 2.** Summary of the stable isotope results from the Mexican turkeys analysed in this paper. The diet is evaluated considering the *δ*^13^C values of each specimen.

		*δ*^13^C_VPDB_(‰)			*δ*^15^N_AIR_(‰)			
geographic area	*n*=	min/max	mean	s.d.	min/max	mean	s.d.	diet
Northern Mexico	7	−17.2/−6.8	−10.0	3.94	7.6/9.8	8.6	0.76	mixed, C_4_
Western Mexico	5	−10.7/−7.8	−9.1	1.22	6.7/8.0	7.4	0.59	C_4_
Gulf Coast	4	−8.5/−6.6	−7.8	0.85	7.5/9.7	8.7	0.93	C_4_
Central Mexican Highlands	17	−18.7/−8.4	−12.3	3.42	3.5/8.9	6.7	1.73	C_3_, mixed, C_4_
Yucatan Peninsula	13	−20.5/−7.2	−14.5	6.06	4.7/9.4	6.8	1.77	C_3_, C_4_

**Figure 3.**
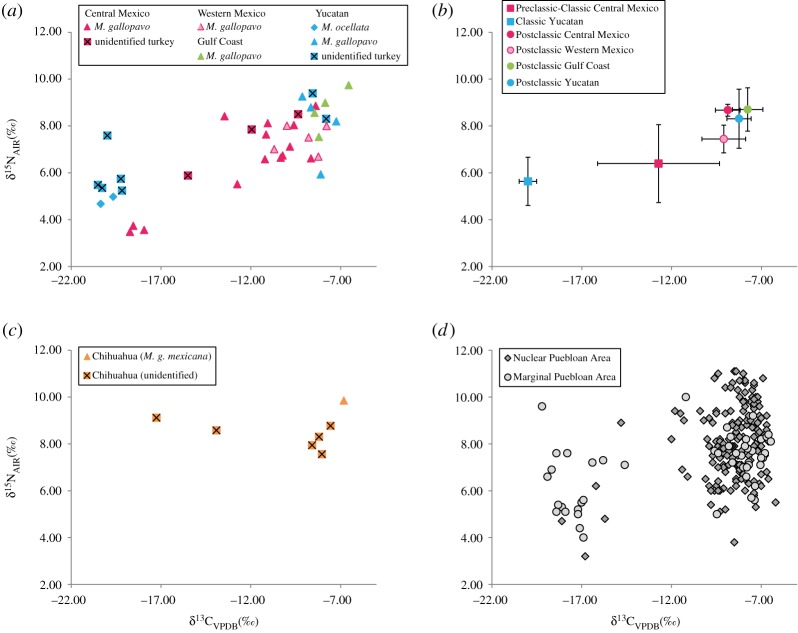
Scatter plots of carbon and nitrogen isotopic values. (*a*) Mesoamerican turkeys analysed in this study, grouped according to site location and genetic identification. (*b*) Means of isotope values for the Mesoamerican turkeys analysed here, according to chronological and geographical distribution; error bars represent 1 s.d. (*c*) Turkeys from Chihuahua, Northern Mexico, analysed in this study. (*d*) Published isotopic data for the nuclear Puebloan region [7,15,17,18] and marginal areas [16] of the SW USA used as comparison with Mexican turkeys.

We observed differences in feeding strategies between regions, time periods and turkey species (figure 3*a*). Within the Maya area (encompassing Yucatan and Northern Central America), turkeys fell within two distinct *δ*^13^C groups, according to their chronology (*U* = 0, *z* = −2.80, *p *= 0.005). Turkeys from the Classic period sites of Calakmul and Chichén Itzá displayed ^13^C-depleted values of −19.2 to −20.5‰ (*n* = 6, mean *δ*^13^C = −20.0 ± 0.48‰); DNA identified specimens at these sites as *M. ocellata*. In contrast, turkeys from the Postclassic site of Champotón displayed ^13^C-enriched values of −7.8 to −9.1‰ (*n* = 6, mean *δ*^13^C = −8.3 ± 0.67‰), indicating diets reliant primarily on C_4_ plants; DNA identified turkeys from this site as *M. gallopavo*. We also observed a significant increase in *δ*^15^N in the Classic compared to the Postclassic groups (*U* = 1, *z*= −2.64, *p *= 0.008). Together, these results support different feeding behaviour in the two turkey species present in the Maya region, consistent with Thornton *et al*.'s [30] recently published isotopic study.

In Central Mexico, the only sample that can be accurately attributed to a Preclassic context (TU206) displays intermediate *δ*^13^C consistent with a mixed feeding ecology. Turkeys from the Classic metropolis of Teotihuacan (*n* = 6) display highly variable isotopic values that seem to cluster in two different groups. Group 1 turkeys (*n* = 3, mean *δ*^13^C = −18.4 ± 0.41‰; mean *δ*^15^N = 3.6 ± 0.13‰) are particularly low in both *δ*^13^C and *δ*^15^N and represent birds with a predominantly C_3_ diet. Group 2 turkeys (*n* = 3, mean *δ*^13^C = −11.1 ± 0.91‰; mean *δ*^15^N = 7.2 ± 0.86‰) display higher *δ*^13^C and *δ*^15^N values and cluster with other Classic–Late Classic birds from the same region (*n *= 8, mean *δ*^13^C = −10.9 ± 1.45‰; mean *δ*^15^N = 7.2 ± 0.49‰) from which they do not differ statistically (*U* = 5, *z* = −0.65, *p *> 0.05). The six samples from Teotihuacan have been attributed to three different haplotypes, mHap1 (*n *= 2) and mHap2/mHap2a (*n *= 4), but there are no differences in the carbon (*U* = 4, *z* = −0.23, *p *> 0.05) or nitrogen values (*U *= 2, *z*=−0.69, *p *> 0.05) that would suggest a direct relation between the genetic lineage and feeding habits.

Postclassic turkeys from Western Mexico and the Gulf Coast display very high *δ*^13^C values ranging from −10.7 to −7.8‰ (mean *δ*^13^C = −9.1 ± 1.22‰) and from −8.5 to −6.6‰ (mean *δ*^13^C = −7.8 ± 0.85‰), respectively, and do not differ statistically (*U* = 4, *z *= −1.35, *p *> 0.05).

We applied stable isotope analysis to nine samples from Chihuahua, Northern Mexico (outside of the Mesoamerican culture area), that were previously tested for aDNA [19]. The seven samples with reliable collagen extraction display a broad variation in *δ*^13^C values (from −17.2 to −6.8‰, mean *δ*^13^C = −10.0 ± 3.94‰), suggesting mixed feeding ecologies as well as diets composed almost exclusively of C_4_ plants. Only two turkey bones from the Calderón site yielded mtDNA haplotypes [19], both of which matched the dominant haplotype observed in the local Gould's wild turkey (*M. g. mexicana*). While isotope values could only be obtained from one of the two DNA identified samples (TU152), it displayed the most enriched *δ*^13^C at the site, indicating that at least some local turkeys had access to large quantities of maize or other C_4_ plants (figure 3*c*).

## 4. Discussion

In this study, we sought to examine how the process of turkey domestication unfolded in Mesoamerica by assessing the origins and genetic diversity of exploited (sub)species or lineages and the extent to which natural turkey feeding behaviours were influenced by humans. Our results indicated that multiple populations of turkeys were exploited in pre-contact Mesoamerica with feeding behaviours that ranged from predominantly (wild) C_3_ plants to those heavily enriched with (cultivated) C_4_ plants. Comparing these results with similar published data from Mesoamerica and SW USA and with zooarchaeological evidence for the presence of turkeys in Mesoamerica, we discuss their implications in terms of understanding the nature of exploited populations, the locations of domestication centres and the varying intensity of human–turkey relationships within pre-contact Mesoamerica.

### 4.1. Exploitation of local populations

Our mtDNA results indicate that at least five populations of turkeys were exploited in North America, with a strong geographical component. In the Central Mexican Highlands, Western Mexico and Gulf Coast, archaeological turkeys displayed mitochondrial lineages identical or closely related to the local South Mexican wild turkey, *M. g. gallopavo* (and Rio Grande wild turkey, *M. g. intermedia*). In Yucatan, the genetic data from this and previous studies [29,30] confirm that both the local ocellated turkeys (*M. ocellata*) and ‘exotic’ common turkeys (*M. gallopavo*) were exploited, with the local species representing only around one-third of the tested assemblage. In Northern Mexico, an intermediate region between the nuclear Puebloan area and Mesoamerica, we observe the exploitation of a third local population, represented by Gould's wild turkey (*M. g. mexicana*).

This pattern of local exploitation contrasts with the SW USA, where the most commonly exploited lineage of turkeys does not appear to be derived from the local wild turkey subspecies (*M. g. merriami*), but instead appears to have been introduced into the region. They form a specific haplogroup (H1) that has no wild equivalent recognized yet. In the SW USA, ‘local/wild’ birds represent a relatively minor component of exploited turkey stocks, making up approximately 15% of tested assemblages even in regions which were thought to support relatively large stocks of wild turkeys [6,19].

### 4.2. North American domestication centres

Our observation of ancient *M.g. gallopavo* haplotypes in Mesoamerican turkey remains is consistent with the historical understanding of Central Mexico as the domestication centre, with the South Mexican wild turkey (*M. g. gallopavo*) as the progenitor subspecies [5,19,49]. The two predominant ancient Mesoamerican haplotypes, mHap1 and mHap2, are also found in Rio Grande wild turkey populations, making this subspecies another potential progenitor for Mesoamerican lineages. Pinpointing the specific geographical origin(s) of the Mesoamerican domestic turkeys is confounded by a scarcity of palaeontological records [9,50] and a lack of modern genetic reference data for Rio Grande populations in Eastern Mexico and for the (presumably extinct) South Mexican wild turkeys in Central Mexico. Archaeological evidence suggests that some of the earliest turkey exploitation in Preclassic periods occurred within the Gulf of Mexico area [51], although these sites are located south of the presumed natural distribution of the wild turkey. Some sites from the central valleys of Oaxaca also display significant numbers of turkey bones from the Preclassic period onwards [52–54]. Genetic and isotopic analyses of turkey bones from these and other early sites may be key to deciphering the origins of domestication in the region.

When assessed within a broader continental context, the ancient Mesoamerican mitochondrial lineages we recovered in this study demonstrate a clear distinction from the indigenous lineage of domestic turkey raised by the ancestral Puebloan of the SW USA, confirming that the two geographic centres were exploiting separate populations of domestic turkeys. These results support the presence of two turkey domestication centres in pre-contact North America, one involving *M. g. gallopavo* (and/or *M. g. intermedia*) in central Mexico and the second (progenitor population as yet unknown) resulting in the domestic breed of the American Southwest. Furthermore, the close relationship between Mexico's archaeological turkeys and modern turkeys [35] reinforces our historical understanding that modern European breeds ultimately originate from central or south-central Mexico rather than elsewhere in North America [49,55]. Lying between these two regions, within Northern Mexico, we see evidence of the exploitation (and likely captive rearing) of a third population, *M. g. mexicana* [19].

The apparent absence of turkey trading between Mesoamerica and the SW USA (at least as demonstrated by mtDNA data) would suggest that turkey domestication occurred as biologically independent events [56], even if the initial domestication centres and specific progenitor populations are not yet accurately identified. The use of distinct turkey populations thus contrasts with numerous examples of trade and exchanges of managed resources between the two regions, including a variety of domestic crops (e.g. [57,58]) and living scarlet macaws [3,59–61]. The extent to which concepts and practices of captive turkey rearing (if not the live birds themselves) were exchanged between the two regions remains to be explored; however, greater resolution in our knowledge of the timings of turkey rearing in both regions may suggest the direction of potential diffusion.

### 4.3. Timing and intensity of human intervention

We assessed the nature and intensity of human intervention within Mesoamerican turkey populations in terms of their reproduction and management through both the genetic and isotopic evidence. The Mexican archaeological turkey samples demonstrate relatively little mitochondrial diversity, with four haplotypes present in the ancient remains. Some archaeological sites (notably the Postclassic sites of Malpais Prieto in Western Mexico, and Huixtoco in Central Mexico) display the presence of only a single haplotype, respectively (mHap1). This genetic uniformity may be indicative of a genetic bottleneck, the reduction in genetic diversity often associated with the limited breeding group of domestic stocks [62], and thus evidence that these bones would represent managed birds, rather than local wild turkeys. This mitochondrial homogeneity is akin to that observed within the domestic populations of the SW USA, where many sites have abundant turkey bones, and strong archaeological evidence for penning and on-site breeding ([4,18], e.g. [63]). However, there is a lack of comparative mtDNA data from Mexican wild turkey populations, making it difficult to assess the range of genetic variation that may have been present in founding or local wild populations. Additional nuclear genetic analyses of modern and historic Mexican wild turkey samples, along with analysis of modern indigenous Mexican turkey breeds may help to clarify the range of mtDNA variation present in wild and domestic remains, and restrictions in genetic diversity and selective sweeps that might have accompanied the domestication process [64].

In contrast to the genetic data, stable isotope ratios of carbon and nitrogen can provide greater resolution in measuring the degree of animal management in ancient North America. Several studies have indicated that captive animals under human control displayed higher values of *δ*^13^C due to reliance on domestic C_4_ crops such as maize, as well as an increase in *δ*^15^N values that could be related with the consumption of manured crops and human food scraps or as an effect of confinement. Similar isotopic shifts between wild and managed animals in North and Central America have been observed in turkeys [7,16,30], leporids [65,66], deer [67], wolves, cougar and golden eagle [68]. In the case of common turkeys, previous isotopic data suggest a strong reliance on maize in both the SW USA and the Maya area [7,15,17,18,30]. It is only in more marginal areas of the American Southwest, where maize was not readily available, that they seem to rely on a mixed C_3_/C_4_ diet, suggesting ‘free-range’ husbandry practices [16]. Here, we consider the chronology and intensity of turkey management as evidenced by the stable isotope analysis.

#### 4.3.1. Preclassic and Classic turkey husbandry

The isotopic results from this and other studies reinforce the variability in turkey husbandry practices throughout Mesoamerica during the Preclassic and Classic periods. The earliest evidence of maize provisioning occurs during the Preclassic period, as revealed by the analysis of the Maya turkeys at El Mirador [30] (*n *= 3, mean *δ*^13^C = −11.8 ± 2.5‰). In contrast, the turkey we analysed from Oaxtepec, Morelos, associated with a Preclassic grave, displays a more mixed diet (*δ*^13^C = −15.5‰) very similar to the turkeys from the marginal region of Gallinas in the American Southwest [16], potentially representing a more free-ranging animal, or a wild bird with access to significant quantities of maize. Together, these data indicate that different degrees of turkey management were emerging in Mesoamerica before the start of the common era, with perhaps greater human intervention occurring in non-local bird populations, such as those in the Maya region.

**Figure 4.**
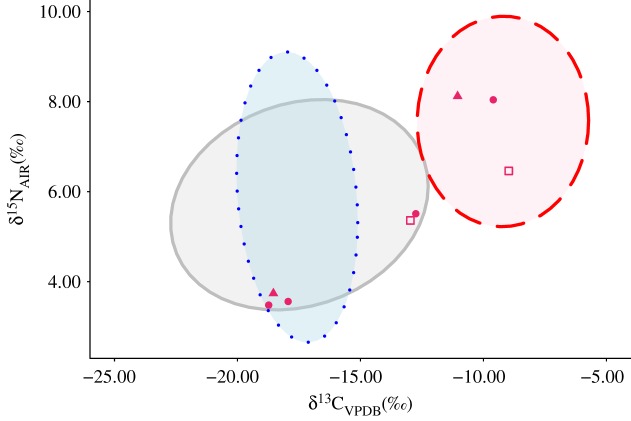
Scatter plot of carbon and nitrogen isotopic values of turkeys from Teotihuacan, from this study (haplotype mHap1, triangle; haplotypes mHap2/mHap2a, circle) and previously published specimens ([69], square). Data compared with ellipses (0.95 confidence interval) calculated for modern wild common turkeys ([7,16,48], plain grey line, *n *= 44), archaeological free-range common turkeys ([16], dotted blue line, *n *= 18) and other archaeological common turkeys from Mesoamerica (this study, [30], dashed red line, *n* = 49).

In the Classic metropolis of Teotihuacan, the six samples analysed here and two samples previously analysed from the Teopancazco district [69] show differential access to C_4_ plants and different levels of enrichment in ^15^N (figure 4). Although our sample size is small, the three individuals with the lowest values of *δ*^13^C show no statistical differences from modern North America wild turkeys [7,16,48] or archaeological free-range raised turkeys [16] (Kruskal–Wallis *χ*^2^ = 1.626, *p *> 0.05), indicating a similar access to wild plants. These turkeys also display extremely low *δ*^15^N values, although there is no significant difference from other Classic and Late Classic remains in the region. Conversely, the other turkeys from Teotihuacan display enriched isotopic values closer to other analysed common turkeys within Mesoamerica. These data suggest that turkeys were subject to varying degrees of management practices in Teotihuacan: half of the birds seem to represent wild turkeys or free-range raised birds, while the others display evidence of a more constrained diet based on mid-to-high quantities of C_4_ plants, certainly provisioned by humans. The presence of both kind of turkeys in similar contexts from Teotihuacan suggests the absence of systematic intensive turkey husbandry practice in the metropolis, an observation reinforced by this bird's secondary rank in importance in terms of human subsistence (as suggested by zooarchaeological data) [70]. Nonetheless, a larger sample size would be necessary to refine these initial findings. In the Maya area, joint genetic and isotopic results arising from this study suggest that only wild ocellated turkeys were exploited during the Classic period, which is consistent with data from earlier work [30].

#### 4.3.2. Postclassic intensification

**Figure 5.**
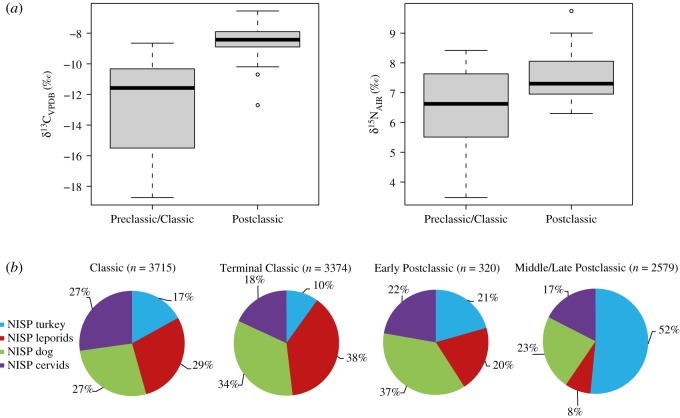
Intensification of common turkey husbandry during the Postclassic period, seen through stable isotope and zooarchaeological data. (*a*) Stable isotope ratios of carbon and nitrogen from this study and Thornton *et al*. [30], showing significant differences between Preclassic/Classic (*n* = 14) and Postclassic (*n *= 30) birds. (*b*) Relative proportions of turkey, leporids, dog and deer in Mesoamerican archaeological corpus from the Early Classic to the Middle/Late Postclassic period, considering the archaeological sites where common turkeys have been identified (electronic supplementary material, table S3).

During the Postclassic period, we observe significantly higher values of both *δ*^13^C (*U* = 26.5, *z *= −4.61, *p *< 0,001) and *δ*^15^N (*U *= 102, *z *= −2.71, *p *= 0.007) (figures 3*b* and 5*a*), suggesting an intensification of management or otherwise increased access of wild birds to human crops. This Postclassic increase in *δ*^13^C demonstrates that turkey diet shifted further away from a wild diet, and that turkeys were relying ever more heavily on provisioned maize or other C_4_ plants, similar to the pattern observed in the SW USA (figure 3*c*) [7,17,18]. *δ*^15^N values may be influenced by a wide range of ecological factors such as the trophic level (as omnivores, turkeys eat a range of protein sources including insects that tend to possess higher *δ*^15^N values) and nutritional stress [71–73]. Other factors depend on the environment, including aridity [74,75], soil salinity [76] or crop manuring [77,78]. While the Postclassic turkeys we analysed here originate from a large variety of environments, from the tropical Maya lowlands to the arid Mexican highlands, their *δ*^15^N values show limited variation (*n* = 30, mean *δ*^15^N = 7.7 ± 1.00‰), suggesting that natural parameters such as climate or edaphic substrate had less impact on nitrogen variations than did the enrichment of soils and crops by human activities (whether the birds were feeding in fertilized human fields, on high nitrogen crops such as beans, or were ingesting crops or prey with nitrogen values inflated by turkey manure) [79,80].

Our isotopic evidence for increased human intervention, provisioning and confinement corresponds with zooarchaeological evidence from the Early Classic to the Postclassic period (figure 5*b*). While turkey bones are rare during the Preclassic period [9], they increase in relative abundance during the Classic period, making up to approximately 40% of the bone remains in Monte Alban [54], although they are still identified in relatively few archaeological sites (electronic supplementary material, table S3). As mentioned above (§4.3.1), common turkeys are conspicuously absent in the Classic Maya sites at this early date. By the Middle/Late Postclassic period (i.e. from 1250 to 1521 CE), the number of turkey bones in archaeological sites overtakes those of leporids, dogs or white-tailed deer (figure 5*b*). Turkeys reappear in the Maya area [30,81], and reach more than 90% in some deposits such as a domestic midden from Texcoco, Central Mexico [82]. However, the motivations behind this intensification in turkey husbandry, particularly in the Middle to Late Postclassic period, are not completely clear. Demographic pressure with increased urbanism at Mesoamerican sites does not seem to be the main motivation for intensification in turkey use, as limited turkey breeding is observed within the metropolis of Teotihuacan, or Classic Maya sites during their florescences [9,30,70].

In the SW USA, the intensification of turkey husbandry has often been explained as a response to a resource depletion [63,83] and this hypothesis can be considered also in Mesoamerica. While the reasons for the collapse of some of the large Maya centres at the end of the Classic period (*ca* 1000 CE) are still debated, environmental changes and droughts have been proposed as factors that may have disrupted the availability of local resources required to support densely populated urban centres (e.g. [84], but see [85] for a discussion). Stable isotopes of carbon and nitrogen from human Maya populations indicate a heterogeneous diet, in particular among the elite (e.g. [86–89]). Although the Postclassic remains analysed are scarce, an overall increase in *δ*^13^C (*n* = 24, mean *δ*^13^C_collagen_ = −9,3 ± 0.8‰) has been interpreted as a dramatic increase in maize consumption at the end of the chronology [89].

A shift from wild C_3_-fed to domestic C_4_-fed animals may also account for the rise in human *δ*^13^C ratios. However, the levels of *δ*^15^N in Postclassic Maya populations (*n* = 24, mean *δ*^15^N = 9.5 ± 0.9‰; data from [86]) are only slightly higher than the Postclassic turkeys’ mean ratio (*n* = 6, mean *δ*^15^N = 8.3 ± 1.3‰) and do not seem to reflect the 3–5‰ spacing expected if turkeys were the main source of protein [90]. The isotopic similarity between Postclassic turkeys and Postclassic human populations suggest similar diets, probably based on high amounts of C_4_-plant proteins, and may alternatively indicate that turkeys were fed with human food scraps, secondarily increasing human *δ*^13^C ratios.

Another compelling argument for the intensification of turkey husbandry in the Postclassic period, and indeed the management of turkeys in early periods in Mesoamerica, relates to the symbolic and political importance of both wild and domestic turkeys throughout the history of Mesoamerica. This role is emphasized by finds such as: the earliest Maya turkeys, all found in ritual caches in elite temples [29]; the more recent finding that all Classic Maya *M. gallopavo* are associated with elite and ritual deposits [30]; a Postclassic palace midden at the site of Texcoco containing almost exclusively turkey bones [82]; and the high frequency of turkey bones in Postclassic funerary deposits at the site of Vista Hermosa (Gulf Coast) and their virtual absence in domestic refuse [91]. Early Spanish observers also mention the use of domestic birds (probably turkeys) to feed captive large carnivores and prey birds [92,93]. In the Aztec capital, ceremonies, feasts and captive carnivore provisioning would have required thousands of turkeys that were sent as tribute [5]. Thornton & Emery [9] also note the importance of increased long-distance trade routes between Central Mexico and the Maya area, as well as expanding trade routes between Mesoamerica, the Gulf Coast of Mexico and the Caribbean during the Postclassic period, all of which may have increased demand and diffusion of turkeys. Thus, unlike the American Southwest, Mesoamerican intensification in turkey husbandry may be less strongly linked to demographic and environmental factors, and may instead have been driven by a strong demand related to the emergence of new policies and trade routes, with the use of domestic birds for various uses including human and carnivore consumption, rituals and sacrifices.

## 5. Conclusion

The intersection of archaeological, DNA and isotopic evidence can provide unprecedented details for documenting the nature, intensity and chronology of human intervention in the evolution of animal species. Our multi-proxy study provides the first biomolecular results confirming that multiple (sub)species of local turkeys were exploited in North America, with genetically distinct domestic lineages raised in the American Southwest and in Mesoamerica. Our Preclassic specimen displayed an intermediate dietary signature, supporting the finding of Thornton *et al*. [29,30] that turkeys were captively reared and provisioned with maize throughout Mesoamerica since the Preclassic period. The increasing frequency of turkey bones in the archaeological record, coupled with evidence of enrichment in carbon and nitrogen isotopic ratios in this study, testifies to the intensification of turkey rearing during the Postclassic period. In contrast to the American Southwest, our data do not conclusively link the intensification of turkey husbandry to demographic or environmental pressures. Further investigations on extended collections are necessary, however, to more fully understand the regional trends of turkey husbandry in relation to cultural development. The mitochondrial genetic data recovered in this study is consistent with other researchers' findings that Mesoamerican birds were the sole progenitors of modern turkey breeds, although whole-genome analyses of heritage turkey breeds and archaeological turkeys may provide further insight into the timing and intensity of selection for desirable traits such as plumage colour, overall size and growth rate [94].

## Supplementary Material

Supplementary Material from Manin et al. “Diversity of Management Strategies in Mesoamerican Turkeys: Archaeological, Isotopic and Genetic Evidence”

## Supplementary Material

Supplementary Table S1 from Manin et al. “Diversity of Management Strategies in Mesoamerican Turkeys: Archaeological, Isotopic and Genetic Evidence”

## Supplementary Material

Supplementary Table S2 and S3 from Manin et al. “Diversity of Management Strategies in Mesoamerican Turkeys: Archaeological, Isotopic and Genetic Evidence”

## Data accessibility

Genetic sequences have been deposited in GenBank under accessions MF947187-MF947219. The isotopic data supporting this article have been uploaded as part of the electronic supplementary material.
