# Supplementary material for: Diversity of management strategies in Mesoamerican turkeys: archaeological, isotopic and genetic evidence
Source: R Soc Open Sci. 2018 Jan 17;5(1):171613. doi: 10.1098/rsos.171613 (PMC5792941; doi:10.1098/rsos.171613)
Supplement: Supplementary Material from Manin et al. “Diversity of Management Strategies in Mesoamerican Turkeys: Archaeological, Isotopic and Genetic Evidence” [file rsos171613supp1.pdf]

Supplementary Material 1

1. Description of the archaeological sites and contexts

Texcoco (TU217): The site of "Los Melones", also known as Ahuehuetitlán, is located in the town of Texcoco, Estado de México, on the southern part of the hill of Los Melones. Rescue excavations lead to the discovery of an high status residential compound that was occupied by the rulers of Texcoco during the 15th century. A total of 143 bones were recovered in domestic middens and platform fillings, from which 97% (139 specimens) were identified as turkey. The bones would have represented 31 individuals of different ages [1]. The specimen analysed in this study comes from this high status Postclassic midden.

Teotihuacan (TU201, TU202, TU203, TU204, TU205, TU211): The archaeological site of Teotihuacan is located in the northeastern part of the Basin of Mexico and recognised by the UNESCO World Heritage. It was one of the largest Mesoamerican city, with more than 100,000 inhabitants estimated at its peak. Extensive excavations and mapping are conducted since the beginning of the 20th century, and show that the city was mainly occupied from the 2nd century BCE until the 7th century CE. During the Classic period (ca. 200-600 CE), Teotihuacan impacted most of the Mesoamerican cultural area [2]. The high level of population density would have required enormous amounts of food with significant implications regarding the environment management [3]. Recent analyses have shown that animal products were mainly acquired through the exploitation of a large range of medium to small animals that would have been easily captured in the cultivated fields or in the nearby lakes [4]. Some of these animals, in particular the leporids, would have also been maintained in captivity in residential compounds, either for human consumption or to feed captive carnivores [5–7]. With at least 167 individuals identified, the turkey represent 6% of the total number of individuals estimated in Teotihuacan [4].

The six turkey remains analysed in this study come from residential compounds of the area known as "La Ciudadela", in the centre. They were found during excavations carried out during the 1980's and deposited in the *Laboratorio de Arqueozoologia M. en. C. Ticul Alvarez*, INAH, but the results of the main zooarchaeological analysis remain unpublished.

Terremote - Tlaltenco (TU209): Terremote - Tlaltenco is located in the south of the Basin of Mexico, on former islands of the Chalco lake. Archaeological excavations carried out in the 1980s indicate that the archaeological site was mainly occupied during the Middle / Late Preclassic (600-200 BCE). It consists in small habitational units showing first evidence of hierarchical structuration. Its inhabitants specialised in the exploitation of lacustrine products, and in particular basketry of the *tule*, a bulrush growing on the shore of the lake [8,9]. Archaeozoological analysis identified the turkey as one of the most abundant animal, together with leporids, deer, anatidae, aquatic turtles and fishes; the turkeys were found in domestic refuses and specific burials [10].

The turkey specimen analysed in this study comes from a domestic context. However, the radiocarbon dating of this sample (327 ± 50 CE) assigns it to a more recent occupation than the main Formative one. The sample is thus considered as a remain dating from the Classic period.

Oaxtepec (TU206): The site is located in the state of Morelos. A rescue archaeology project associated with a highway construction lead to the discovery of a pyramidal basement and a cist containing the primary burial of a young woman in semi-flexed position with ceramic offerings attributed to Middle Preclassic (1200-500 BCE). Out of the cist but still in close association, the skeleton of a complete turkey was found, representing one of the earliest evidence of its symbolic use [11 and references herein]

Xochicalco (TU218, TU219, TU220, TU221, TU222): Xochicalco is a hilltop city located in the eastern part of the state of Morelos, recognised as a UNESCO World Heritage site. While the site was occupied between 900 BCE until 1521 CE, the city reached its height during the Terminal Classic (650-900 CE) gathering 10,000 to 15,000 inhabitants. During this transitional period between Classic and Postclassic, also known as Epiclassic, Xochicalco reached a position of regional capital [12]. Dog, white-tailed deer and puma were the most prevalent species in the faunal collection, while turkey was the most abundant among the birds. Its remains were found in several domestic contexts from both elite and commoners [13]. Given the abundance of domestic animals in some middens, it has been suggested that husbandry was a common activity in Xochicalco, probably practiced at the household scale or as a communal labour [12–14].

The turkey remains analysed in this study are associated with the Terminal Classic occupation, which is confirmed by the radiocarbon dating of one sample (TU221, 817 ± 37 CE). One of the

samples (TU221) was recovered on Loma Sur, in a modest domestic household, whereas the others (TU218, TU219, TU220, TU222) were found in elite households.

Huixtoco, Ixtapaluca (TU207, TU208, TU213, TU215): This site is located in the southern part of the Basin of Mexico, on the shore of former Chalco lake. It was characterised as a small village occupied during the Preclassic period [15]. More recently, rescue excavations driven by the urban growth of Mexico City have yielded animal remains associated with this pre-Hispanic occupation including dog, white-tailed deer and turkey [10,16].

The turkey samples analysed in this study come from these recent excavations. However, the radiocarbon dating of one of the samples (TU213, 410 ± 9 CE) is related with the Classic period. It indicate a potential continuity of the occupation, and the samples are considered as Preclassic/Classic.

Santa Ana Teloxtoc (TU216): The archaeological site is a cave located in the municipality of Tehuacán, in the state of Puebla. Thanks to the dry climatic conditions, this region is known to have yielded exceptionally well preserved biological remains. In the cave of Santa Ana Teloxtoc, wood masks with turquoise inlays, obsidian blades and gourd bowls were found as part of an offering. Other elements were dispersed on the floor, including animal bones and ceramic sherds. Elements characteristics of the Classic period (ceramic sherds) to the Postclassic period (masks) indicate the recurrent use of the cave for ritual purposes [17].

Turkey is the predominant species identified in the cave with 70 bone remains representing at least 9 individuals [18]. Considering the context, they might have been used as offering or for other ritual purpose [17]. However further investigations are needed to to clarify their chronological attribution.

El Tigre (TU212): The site of El Tigre is located in near the La Candelaria river, in the municipality of El Carmen, state of Campeche and its original Maya name was Itzamkanac. During Late Classic (700-100 CE), the city practised trade and exchanges by riverine transportation. The site comprises the main town with ceremonial and administrative areas, surrounded by several smaller villages. In the ceremonial centre, four large buildings as well as a ballcourt, roads and channels were discovered. The faunal remains were recovered from Structure 1 (a residential complex of high status, probably a palace) and Structure 4 (a large platform supporting several mounds), associated with multiple constructive stages. The animal

bones were interpreted as domestic waste, presumably from elite households. They pertains mostly to white-tailed deer, turtles and dogs, as well as monkeys, small carnivores, pecari, ocellated turkey, crocodiles and marine fishes in a lesser extent [19,20].

The specimen analysed in this study was recovered from Structure 1. It could not be DNA identified but stable isotopes indicate its diet was essentially composed of C_3 plants. According to this study and previous results from the Maya area [21], it confirms it might have been a wild ocellated turkey.

Calakmul (TU225, TU226, TU227, TU228, TU229): The archaeological city of Calakmul, recently recognised as a UNESCO World Heritage site, is located in the southeast of the state of Campeche, where long-term archaeological excavations were carried out [22]. Occupied from the Middle Preclassic (600-300 BCE) to the Early Postclassic (800-950 CE), the city was one of the main political centre of the Mayan area during the Late Classic (600-800 CE), gathering around 50,000 inhabitants. The importance of the site is reflected in the architecture and also in evidences of their military and political power in the Maya region [23,24]. Like many other large Maya centres, Calakmul suffered a dramatic decline of population during the Late Classic, where the use of many buildings was changed. Formerly sacred structures were modified to serve as places for quotidian activities, combining residential, administrative, and economic functions [22].

The bone remains analysed in this study were found in Structure II, a structure that switched from a civico-ceremonial building to a residential unit for common people. The remains, deposited in the *Taller de Zooarqueología* at the UADY, were analysed under the direction of Christopher Götz and reported in García Lara [25]. From the five samples analysed here, two were identified as ocellated turkey and had a diet based on C_3 plants; other two could not be genetically identified, but stable isotopes indicate a similar diet, based on wild plants. Thus there is no evidence of the common turkey during the Late Classic occupation.

Champoton (TU230, TU231, TU232,TU233, TU234, TU235): Champoton is both a city and a region. The city is located near the mouth of Rio Champoton, in the state of Campeche. This was a strategic location for the control of coastal and inland trade, providing chances to develop a variety of subsistence strategies over maritime, estuarine or continental resources. The excavations carried out by William Folan and colleagues from the Universidad Autonoma de

Campeche, documents occupations extending from the Middle Preclassic to the Late Postclassic [26]. A large diversity of animals were identified. Among the mammals, dog and white-tailed deer are predominant while among the birds the turkeys and aquatic birds are the most abundants. Reptiles and fishes were also identified [27].

The turkey bones analysed in this study were recovered from a midden of the Postclassic occupation (1200-1500 CE). While the zooarchaeological analysis suggested the presence of both species of turkey, we only identified the common turkey (four specimens on the six analysed here). Moreover, all the samples display high levels of ^{13}C consistent with an diet based on cultivated C_4 plants such as maize.

Chichen Itza (TU223, TU224): The city of Chichen Itza is located in Central Yucatan and recognised as a UNESCO World Heritage site. The presence of mixed architectural and iconographic elements inherited from both the Mayas and the Toltecs (generally limited to the Central Mexican Highlands) raised questions about the origin of this city and its connexion with Northern areas [28]. Based on radiocarbon dates, ceramic, and lithic evidence, the site was occupied between Terminal Classic and Early Postclassic (800-1050 CE). It was a regional centre in the north of the Yucatán Peninsula with trade interactions recognised until Central America, the Gulf Coast, and the Mexican Highlands [29]. Animal bones were recovered from the middens of elite households in the main centre, near the “Galería de los monos”. Mammals represent a large majority of the sample (94%) and the other 6% is composed of birds, reptiles and fishes. Among the birds, ocellated turkey and another large galliform, the great curassow *(Crax rubra)* were identified [30].

The two turkey bones analysed here could not be genetically identified but the stable isotope values are consistent with a diet predominantly composed of of C_3 plants.

Malpais Prieto (TU304, TU305, TU306, TU307, TU308): Malpais Prieto is a hilltop site located in the Basin of Zacapu, in the northern part of the state of Michoacan, gathering around 5,000 inhabitants. The construction of the city is seen as the result of a sudden demographic increase in the Basin, happening around 1250 CE. But by 1400 CE, the site is abandoned. Archaeological excavations indicate that domestic subsistence was mainly based on local resources. Animal products were acquired through the exploitation of commensal and domestic

animals, such as turkeys, cottontails and pocket gophers, while deer hunting seems limited to ritual and funerary contexts [31].

The five turkey bones analysed in this study come from a domestic midden associated with an elite household.

Vista Hermosa (TU302, TU303, TU309, TU310, TU311, TU312, TU313, TU314): Vista Hermosa is located in the south of the state of Tamaulipas, in the Panuco Basin, near the modern town of Nuevo Morelos. The site consists in around 100 architectural structures, mainly rectangular and circular platforms as well as a ball game, occupied between the 14th century and the Spanish Conquest, around 1523 [32]. A total of 116 sepultures were discovered during the archaeological excavations (1965-1967), 35 of them associated with animal remains. Complete skeletons of turkeys were identified in 16 graves, with one to three birds per burial. It makes this bird the most common animal found in funerary context. Turkeys were also present in domestic refuses, although the number of deer and dog bones was superior [33].

The eight turkey specimens analysed in this study all come from funerary deposits.

El Calderon (TU135, TU147, TU148, TU149, TU142, TU250, TU251): El Calderon site (CH-254) is located in Chihuahua, Mexico, in the southern Casas Grandes zone. It was occupied mainly during the Viejo period (800-1250 CE) and spreads on 2 ha of a 8 m high terrace on the west bank of a tributary of rio Santa Maria, the arroyo Teseachic-El Pino. A variety of continental mammals, birds, reptiles and fishes were identified [34].

Turkey bones were found in Structure 2 and Structure 4, as part of domestic middens. Stable isotopes of carbon and nitrogen on human remains from the Calderon site indicate a diet mainly based on C_4 plants with a limited consumption of meat. A previous analysis of a turkey bone show a diet similar to humans, indicating the birds may have been fed with human food scraps, maybe by frequenting the middens around the settlement [35].

2. Supplementary Methods

2.1 Radiocarbon dating

Each bone was cleaned by wire brush and washed, using ultrasonic bath. The crushed bone was treated with 1N HCl at 4° for 24 hours. The residue was filtered, rinsed with deionized water. To dissolve the collagen, the sample was heated at 80ºC for 6 hours at pH=3. The collagen solution was then filtered to isolate pure collagen. The collagen was first dried, then combusted at 575ºC in evacuated/sealed Pyrex ampoule in the present CuO. The resulting carbon dioxide was cryogenically purified from the other reaction products and catalytically converted to graphite using the method of [36]. Graphite 14C/13C ratios were measured using the CAIS 0.5 MeV accelerator mass spectrometer. The sample ratios were compared to the ratio measured from the Oxalic Acid I (NBS SRM 4990). The sample $^{13}C/^{12}C$ ratios were measured separately using a stable isotope ratio mass spectrometer and expressed as $\delta^{13}C$ with respect to PDB, with an error of less than 0.1‰.

Results from radiocarbon dating (Table S2) indicate that most of the elements are attributed to the Classic period (200-900 CE) and one to the Postclassic period (900-1520 CE). These results are consistent with the archaeological expectations, except for two settlements, Huixtoco and Terremote. In these two sites, chrono-stratigraphic elements suggested a Preclassic occupation (650-250 BCE) whereas the two turkey bones that were dated are more recent (410 CE ± 9 for Huixtoco and 327 CE ± 50 for Terremote). These results indicate potential disturbance in the stratigraphic sequence and imply to attribute the other bones from the same settlements to a larger time range, ranging from Preclassic to early Classic.

2.2 Stable Isotope Analysis

The laboratories at the University of British Columbia (UBC) and the University of York (BioArCh) used the same extraction procedure. Collagen was extracted from approximately 300-500 mg of whole bone, following the procedures outlined in Richards and Hedges [37] with the addition of an ultrafiltration step [38]. Samples were demineralised in 0.5M HCl at 5 ºC for 1-5 days, then rinsed in deionised water. The resultant solid was then gelatinised at 70 ºC for 24 - 48 hours and the solution ultrafiltered (30kDa Amicon® Ultra-4 centrifugal filter units; Millipore, Billerica, MA, USA). The retentate was frozen and lyophilised.

Carbon and nitrogen isotope values from bulk collagen were analysed by EA/IRMS. At UBC, collagen (0.5 mg) was measured in duplicate using an Elementar EA coupled to an IsoPrime 100 isotope ratio mass spectrometer. At the University of York, collagen samples (1 mg ± 0.1) were analysed in duplicate in a GSL analyser coupled to a 20-22 mass spectrometer (Sercon, Crewe, UK). For both instruments, analytical error (calculated from repeated measurements of each sample and respective in-house standards) was < 0.2 ‰ (1σ).

2.3 Ancient DNA (aDNA) analysis

aDNA extraction: Forty-eight archaeological turkey bones were analysed in three ancient DNA laboratories (SI Table 1), located in the Department of Archaeology, Simon Fraser University (SFU), the Department of Anthropology and Archaeology, University of Calgary (UofC), and BioArCh, University of York (UofY). In all three laboratories, sample preparation and DNA extraction followed the silica spin-column protocol [39] as described in Speller et al. [40]. Briefly, turkey bones were subsampled using a sterile saw blade, and chemically decontaminated through immersion on 6% sodium hypochlorite for 5-7 min, and rinsed two times in HPLC grade water. Bones samples were UV irradiated for 30 min on two sides before being crushed into powder, and incubated overnight at 50℃ in 2-4ml of lysis (0.5 mg/ml proteinase K, 0.5M EDTA, pH 8). Samples were centrifuged and the supernatant concentrated to 100ul in an Amicon centrifugal filter, Ultra-4 (Millipore) and purified using either a Qiagen Nucleotide Removal Kit or PCR purification Kit.

Amplification: Samples were PCR amplified using primers described in Speller et al. [40] and Thornton et al. [41] with PCR conditions described in Speller et al. [40] to amplify a maximum of 598 bp from the hypervariable control region (D-loop) of the turkey mtDNA genome. Initial PCR reactions were prepared using the longest primer sets (i.e. TK-F2/TK-R405, TK-F315/TK-R670) to test for overall DNA preservation. Samples were also amplified and sequenced using shorter primer sets. Five µL of PCR product were separated by electrophoresis on 2% agarose gel and visualized using SYBR-Green or SYBR-Safe. PCR products and were sequenced using both forward and reverse primers at Eurofins MWG Operon, Alabama. Repeat extractions and amplification of 10 samples were performed in separate laboratories (Table SI).

mtDNA analysis: The obtained ancient DNA sequences were BLAST-compared through GenBank to determine if they would match *Meleagris* sequences, and to ensure that they did not

match with any other unexpected species or sequences. Sequences were visually examined and multiple sequences from the same bone were compiled into consensus sequences using ChromasPro software (www.technelysium.com.au). The complete consensus D-loop sequence for each sample was truncated to 438 bp (position 15567-16004, based on complete mtDNA genome of GenBank specimen NC010195) to remove primer sequences, and make them comparable to reference sequences found in Mock et al. [42] and Speller et al. [40]. The obtained D-loop haplotypes were compared with 298 *Meleagris* GenBank entries, including modern commercial breeds [43] and North American wild turkeys [42,44] as well as the 12 ancient haplotypes identified in the archaeological turkey remains recovered from the American Southwest [40]. Multiple alignments of the haplotypes sequences and published *Meleagris* mtDNA reference sequences were conducted using ClustalW [45] through BioEdit [46]. Median-joining networks were created using Network (v. 5.0) and Network Publisher [47].

Contamination control: The authenticity of the analyzed data was secured by multiple criteria, including: (i) the use of dedicated ancient DNA facilities; (ii) a vigorous decontamination protocol of the bone samples before DNA extraction; (iii) the inclusions of blank extracts and PCR negative controls; (iv) multiple haplotypes were obtained within the study as a whole, as well as within most extraction batches; (v) all sequences indicate the bones were members of the *Meleagris* genus, which is consistent with the morphological identifications. Repeat amplification and sequencing was conducted for all samples, with repeat extraction and amplification of 10 samples in an independent laboratory. Consistent results were obtained for all replications.

SI References

1. Valadez Azúa R, García Chavez R, Rodríguez Galicia B, Gamboa Cabezas L. 2001 Los guajolotes y la alimentación prehispánica. *Cienc. Desarro.* **27**, 55–63.

2. Cowgill GL. 2015 *Ancient Teotihuacan*. Cambridge University Press.

3. Sugiyama N, Somerville AD. 2017 Feeding Teotihuacan: integrating approaches to studying food and foodways of the ancient metropolis. *Archaeol. Anthropol. Sci.* **9**, 1–10. (doi:10.1007/s12520-016-0419-8)

4. Sugiyama N, Azúa RV, Galicia BR. 2017 Faunal acquisition, maintenance, and

consumption: how the Teotihuacanos got their meat. *Archaeol. Anthropol. Sci.* **9**, 61–81. (doi:10.1007/s12520-016-0387-z)

5. Somerville AD, Sugiyama N, Manzanilla LR, Schoeninger MJ. 2016 Animal Management at the Ancient Metropolis of Teotihuacan, Mexico: Stable Isotope Analysis of Leporid (Cottontail and Jackrabbit) Bone Mineral. *PLoS One* **11**, e0159982. (doi:10.1371/journal.pone.0159982)

6. Somerville AD, Sugiyama N, Manzanilla LR, Schoeninger MJ. 2017 Leporid management and specialized food production at Teotihuacan: stable isotope data from cottontail and jackrabbit bone collagen. *Archaeol. Anthropol. Sci.* **9**, 83–97. (doi:10.1007/s12520-016-0420-2)

7. Sugiyama N, Somerville AD, Schoeninger MJ. 2015 Stable Isotopes and Zooarchaeology at Teotihuacan, Mexico Reveal Earliest Evidence of Wild Carnivore Management in Mesoamerica. *PLoS One* **10**, e0135635. (doi:10.1371/journal.pone.0135635)

8. Serra Puche MC, Lazcano Arce JC. 2009 Arqueología en el sur de la cuenca de México. Diagnóstico y futuro: In memoriam WT Sanders. *Cuicuilco* **16**, 19–38.

9. Serra Puche MC. 1988 *Los recursos lacustres de la cuenca de México durante el Formativo*. México: Coordinación General de Estudios de Posgrado.

10. Valadez Azúa R, Rodríguez Galicia B. 2014 Uso de la fauna, estudios arqueozoológicos y tendencias alimentarias en culturas prehispánicas del centro de México. *Anales de antropología* **48**, 139–166.

11. Corona-M E. 2006 Una ofrenda de guajolote en el sitio Oaxtepec km 27.5, Morelos. In *Memorias del IV Congreso Interno del Centro INAH Morelos* (eds AG Canto, GL Ledesma, GM Tostado, MM Fuentes, FJ Nau, MM Morayta), pp. 49–52. México: Instituto Nacional de Antropología e Historia.

12. Hirth KG. 2000 *Archaeological research at Xochicalco*. The University of Utah Press.

13. Corona-M E. 2008 Zoogeographical affinities and the use of vertebrates in Xochicalco, Morelos, Mexico. *Quat. Int.* **180**, 145–151. (doi:10.1016/j.quaint.2007.09.040)

14. Heath-Smith C. 2000 Analysis of faunal material recovered from Xochicalco. In *Archaeological research at Xochicalco* (ed KG Hirth), pp. 184–192. University of Utah Press.

15. Parsons JR, Brumfiel E, Parsons MH, Wilson DJ. 1982 Prehispanic Settlement Patterns in the Southern Valley of Mexico: The Chalco-Xochimilco Region. Memoirs No. 14. Museum of Anthropology, University of Michigan. *Ann Arbor*

16. Valadez R, Gamboa L, Vélez N, Rodríguez B, Gómez M, García R, Pérez G. 2004 Perros y prácticas rituales en una antigua aldea de la cuenca de México. *AMMVEPE* **15**, 158–171.

17. Vargas Pacheco E. 1989 *Las máscaras de la cueva de Santa Ana Teloxtoc*. Mexico: Universidad Nacional Autonoma de México.

18. Valadez R. 1989 Restos animales. In *Las máscaras de la cueva de Santa Ana Teloxtoc* (ed E Vargas), pp. 142–150. Mexico: Universidad Nacional Autonoma de México.

19. Valadez R, Rodríguez B, Gómez M. 2013 Paleozoologia. In *Itzamkanac, El Tigre, Campeche: exploración, consolidación y análisis de los materiales de la Estructura 1* (ed E Vargas Pacheco), pp. 355–380. Mexico: Universidad Nacional Autónoma de México, Instituto de Investigaciones Antropológicas, Gobierno del Estado de Campeche, Secretaría de Cultura de Campeche, CONACULTA.

20. Vargas Pacheco E. 2013 *Itzamkanac, El Tigre, Campeche: exploración, consolidación y análisis de los materiales de la Estructura 1*. Universidad Nacional Autónoma de México, Instituto de Investigaciones Antropológicas, Gobierno del Estado de Campeche, Secretaría de Cultura de Campeche, CONACULTA.

21. Thornton E, Emery KF, Speller C. 2016 Ancient Maya turkey husbandry: Testing theories through stable isotope analysis. *Journal of Archaeological Science: Reports* **10**, 584–595. (doi:10.1016/j.jasrep.2016.05.011)

22. Braswell GE, Gunn JD, Carrasco, M. D. R. D.: Folan, W. J., Fletcher LA, López AM, Glascock MD. 2004 Defining the terminal classic at Calakmul. In *The terminal Classic in the maya lowlands: Collapse, transition, and transformation* (eds AA Demarest, PM Rice, DS Rice), Boulder: University Press of Colorado.

23. Folan WJ, Marcus J, Pincemin S, del Rosario Domínguez Carrasco M, Fletcher L, López AM. 1995 Calakmul: New Data from an Ancient Maya Capital in Campeche, Mexico. *Latin American Antiquity* **6**, 310–334. (doi:10.2307/971834)

24. del Rosario Domínguez Carrasco M. 1994 Tipología cerámica de Calakmul, Campeche, México. *Mexicon* **16**, 51–53.

25. García Lara SL. 2008 El uso de animales en las Tierras Bajas Mayas Centrales. Un estudio de los restos de fauna recuperados en la estructura II de Calakmul, Campeche.

26. Ek J. 2012 The political and economic organization of Late Classic states in the Peninsular Gul coast: the view from Champoton, Campeche. In *The ancient Maya of Mexico: Reinterpreting the past of the northern Maya lowlands* (ed GE Braswell), pp. 143–169. Sheffield: Equinox Press.

27. Götz CM. 2008 Coastal and inland patterns of faunal exploitation in the prehispanic northern Maya lowlands. *Quat. Int.* **191**, 154–169. (doi:10.1016/j.quaint.2008.02.003)

28. Kowalski Jeff Karl. 2007 *Twin Tollans: Chichén Itzá, Tula, and the Epiclassic to Early Postclassic Mesoamerican World*. Dumbarton Oaks.

29. Cobos R. 1997 Chichen Itza y el Clásico Terminal en las Tierras Bajas Mayas. In *XI*

Simposio de Investigaciones Arqueológicas en Guatemala (eds JP Laporte, H Escobedo), pp. 915–930. Guatemala: Museo Nacional de Arqueología y Etnología.

30. Götz C. 2008 Manjares del pasado: contrastes del aprovechamiento faunístico entre sitios prehispánicos costeros y de tierra adentro de las Tierras bajas del Norte. In *XXI Simposio de Investigaciones Arqueológicas en Guatemala* (eds JP Laporte, B Arroyo, H Escobedo, H Mejia), pp. 781–798. Guatemala: Museo Nacional de Arqueología y Etnología.

31. Manin A, Pereira G, Lefèvre C. 2015 El uso de los animales en una ciudad tarasca posclásica: estudio arqueozoológico del sitio de Malpaís Prieto (Michoacán, Mexico). *Archaeobios* **1**, 28–42.

32. Stresser-Péan G. 1975 Fouilles à Vista Hermosa, municipio de Nuevo Morelos, Tamaulipas (Huasteca). In *Actas del 41 Congreso Internacional de Americanistas*, pp. 347–352. México: Comisión de Publicación de las Actas y Memorias.

33. Manin A, Lefèvre C. 2018 Uso material y simbólico de los animales en Vista Hermosa. In *Vista Hermosa. Nobles, artesanos y mercaderes en los confines del mundo huasteco. Estudio arqueológico de un sitio posclásico tardio del municipio de Nuevo Morelos, Tamaulipas, México* (eds C Stresser-Péan, G Pereira, G Stresser-Péan), vol.3. Mexico D.F.: Secretaría de Cultura, Instituto Nacional de Antropología e Historia, Museo Nacional de Antropología, Fundación Stresser-Péan, Centro de Estudios Mexicanos y Centroamericanos.

34. Kelley JH, Garvin RD, Stewart JD, Zbrover D, Chiykowski T, Adams KR. 2014 *The Viejo Period in West-central Chihuahua: The Calderón Site*. University of New Mexico, Maxwell Museum of Anthroppology.

35. Webster M, Katzenberg MA. 2008 Dietary reconstruction and human adaptation in west central Chihuahua. In *Celebrating Jane Holden Kelley and her work* (ed K Meade), pp. 71–102. Albuquerque: New Mexico Archaeological Council.

36. Vogel JS, Southon JR, Nelson DE, Brown TA. 1984 Performance of catalytically condensed carbon for use in accelerator mass spectrometry. *Nucl. Instrum. Methods Phys. Res. B* **5**, 289–293. (doi:10.1016/0168-583X(84)90529-9)

37. Richards MP, Hedges REM. 1999 Stable Isotope Evidence for Similarities in the Types of Marine Foods Used by Late Mesolithic Humans at Sites Along the Atlantic Coast of Europe. *J. Archaeol. Sci.* **26**, 717–722. (doi:10.1006/jasc.1998.0387)

38. Brown TA, Nelson DE, Vogel JS, Southon JR. 1988 Improved collagen extraction by modified Longin method. *Radiocarbon* **30**, 171–177. (doi:10.2458/azu_js_rc.30.1096)

39. Yang DY, Eng B, Waye JS, Dudar JC, Saunders SR. 1998 Technical note: improved DNA extraction from ancient bones using silica-based spin columns. *Am. J. Phys. Anthropol.* **105**, 539–543. (doi:3.0.CO;2-1">10.1002/(SICI)1096-8644(199804)105:4<539::AID-AJPA10>3.0.CO;2-1)

40. Speller CF, Kemp BM, Wyatt SD, Monroe C, Lipe WD, Arndt UM, Yang DY. 2010 Ancient mitochondrial DNA analysis reveals complexity of indigenous North American turkey domestication. *Proceedings of the National Academy of Sciences* **107**, 2807–2812. (doi:10.1073/pnas.0909724107)

41. Thornton EK, Emery KF, Steadman DW, Speller C, Matheny R, Yang D. 2012 Earliest Mexican Turkeys (Meleagris gallopavo) in the Maya Region: Implications for Pre-Hispanic Animal Trade and the Timing of Turkey Domestication. *PLoS One* **7**, e42630. (doi:10.1371/journal.pone.0042630)

42. Mock KE, Theimer TC, Rhodes OE, Greenberg DL, Keim P. 2002 Genetic variation across the historical range of the wild turkey (Meleagris gallopavo). *Mol. Ecol.* **11**, 643–657.

43. Monteagudo LV, Avellanet R, Azón R, Tejedor MT. 2013 Mitochondrial DNA analysis in two heritage European breeds confirms Mesoamerican origin and low genetic variability of domestic turkey. *Anim. Genet.* **44**, 786. (doi:10.1111/age.12080)

44. Szalanski AL, Church K, Oates D, Bischof R, Powers TO. 2000 Mitochondrial DNA variation within and among wild turkey (Meleagris gallopavo) subspecies. *Trans. Nebr. Acad. Sci.* **26**, 47–53.

45. Thompson JD, Higgins DG, Gibson TJ. 1994 CLUSTAL W: improving the sensitivity of progressive multiple sequence alignment through sequence weighting, position-specific gap penalties and weight matrix choice. *Nucleic Acids Res.* **22**, 4673–4680.

46. Hall TA. 1999 BioEdit: a user-friendly biological sequence alignment editor and analysis program for Windows 95/98/NT. *Nucleic Acids Symp. Ser.* **41**, 95–98.

47. Bandelt H-J, Forster P, Roehl A. 1999 Median-joining networks for inferring intraspecific phylogenies. *Mol. Biol. Evol.* **16**, 37–48.
