# Supplementary material for: Diversity of management strategies in Mesoamerican turkeys: archaeological, isotopic and genetic evidence
Source: R Soc Open Sci. 2018 Jan 17;5(1):171613. doi: 10.1098/rsos.171613 (PMC5792941; doi:10.1098/rsos.171613)
Supplement: Supplementary Table S2 and S3 from Manin et al. “Diversity of Management Strategies in Mesoamerican Turkeys: Archaeological, Isotopic and Genetic Evidence” [file rsos171613supp3.docx]

**Table S2: Results of radiocarbon dating from bone collagen**

| Sample ID | 14C age  (years BP) | ± | pMC | ± | Calibrated date |
| --- | --- | --- | --- | --- | --- |
| TU201 | 1780 | 20 | 80.14 | 0.23 | 261 CE ± 40 |
| TU213 | 2360 | 20 | 74.51 | 0.22 | 410 CE ± 9 |
| TU209 | 1700 | 20 | 80.93 | 0.23 | 327 CE ± 50 |
| TU221 | 1210 | 20 | 86.04 | 0.25 | 817 CE ± 37 |
| TU233 | 660 | 20 | 92.06 | 0.25 | 1331 CE ± 42 |

Note: The ^14^C dates have been corrected for isotope fractionation;error margins are one standard deviation, and reflect both statistical and experimental errors.

**Table S3: Abundance of turkey remains and other fauna in Mesoamerican Archaeological sites**

| **Chronology (AD)** | **Site** | **Turkey** | **Leporids** | **Dog** | **Cervids** | **References** |
| --- | --- | --- | --- | --- | --- | --- |
| Classic (180-650) | Teotihuacan | 167* | 684* | 326* | 311* | Sugiyama et al. (2016) |
|  | JR74 | 4 | 27 | 32 | 110 | Manin & Lefèvre (2016) |
|  | El Palmillo (C) | 33 | 233 | 357 | 280 | Lapham et al. (2013) |
|  | Mitla Fortress (C) | 18 | 20 | 36 | 6 | Lapham et al. (2013) |
|  | Monte Alban | 408 | 102 | 257 | 304 | Martinez Lira (2014) |
| Late Classic (650-800) | Xochicalco | 31 | 10 | 5 | 4 | Heath-Smith (2000) |
|  | Santa Cruz Atizapan | 26* | 28* | 87* | 75* | Valadez Azua & Rodriguez Galicia (2009) |
|  | El Palmillo (E) | 167 | 1160 | 835 | 435 | Lapham et al. (2013) |
|  | Mitla Fortress (E) | 118 | 84 | 175 | 61 | Lapham et al. (2013) |
|  | Tepetitlan | 2 | 3 | 34 | 34 | Polaco (1999) |
| Early Postclassic (900-1200) | El Canal/El Coral | 1^✢^ | 16^✢^ | 24^✢^ | 45^✢^ | Diehl (1981) |
|  | Mitla Fortress (PC) | 30 | 14 | 36 | 11 | Lapham et al. (2013) |
|  | Chalco | 35 | 35 | 58 | 15 | Guzman & Polaco (2008) |
| Middle/Late Postclassic (1200-1521) | Calixtlahuaca | 26 | 8 | 88 | 23 | Manin & Lefèvre (2016) |
|  | Malpais Prieto | 246 | 137 | 2 | 157 | Manin & Lefèvre (2016) |
|  | Yautepec | 2 | 1 | 9 | 22 | Wharton & Smith (2006) |
|  | Capilco | 80 | 17 | 230 | 1 | Heath-Smith & Wharton (2006) |
|  | Cuexcomate | 5 | 5 | 18 | 0 | Heath-Smith & Wharton (2006) |
|  | Vista Hermosa | 651 | 12 | 69 | 140 | Manin & Lefèvre (2017) |
|  | Texcoco | 139 | 0 | 1 | 2 | Valadez Azua et al. (2001) |
|  | Mayapan | 112* | 18* | 48* | 52* | Mason & Peraza Lopez (2013) |
|  | Champoton | 63 | 6 | 124 | 65 | Götz (2008) |

Note: *Published data only included MNI; ✢Published data only gave a percentage of the total assemblage
